# Using machine learning to explore core risk factors associated with the risk of eating disorders among non-clinical young women in China: A decision-tree classification analysis

**DOI:** 10.1186/s40337-022-00545-6

**Published:** 2022-02-10

**Authors:** Yaoxiang Ren, Chaoyi Lu, Han Yang, Qianyue Ma, Wesley R. Barnhart, Jianjun Zhou, Jinbo He

**Affiliations:** 1 School of Humanities and Social Science, The Chinese University of Hong Kong, Shenzhen, 518172 Guangdong China; 2 School of Data Science, The Chinese University of Hong Kong, Shenzhen, Guangdong China; 3 Present Address: Shenzhen Research Institute of Big Data, Shenzhen, Guangdong China; 4 Department of Psychology, Bowling Green State University, Bowling Green, Ohio, USA

**Keywords:** Eating disorders, Machine learning, Decision tree, Risk factors, Chinese women

## Abstract

**Background:**

Many previous studies have investigated the risk factors associated with eating disorders (EDs) from the perspective of emotion regulation (ER). However, limited research has investigated interactions between co-existing risk factors for EDs, especially in China where research in EDs is underrepresented.

**Methods:**

This study examined core risk factors related to maladaptive eating behaviors and ER, and how their interactions affect the detection of EDs. Using machine learning, a decision tree model was constructed on a data set of 830 non-clinical Chinese young women with an average age of 18.91 years (*SD* = 0.95). The total data set was split into training and testing data sets with a ratio of 70 to 30%.

**Results:**

Body image inflexibility was identified as the major classifier for women at high risk of EDs. Furthermore, interactions between body image inflexibility, psychological distress, and body dissatisfaction were important in detecting women at high risk of EDs. Overall, the model classifying women at high-risk for EDs had a sensitivity of 0.88 and a specificity of 0.85 when applied to the testing data set.

**Conclusions:**

Body image inflexibility, psychological distress, and body dissatisfaction were identified as the major classifiers for young women in China at high risk of EDs. Researchers and practitioners may consider these findings in the screening, prevention, and treatment of EDs among young women in China.

**Supplementary Information:**

The online version contains supplementary material available at 10.1186/s40337-022-00545-6.

## Plain English summary

Previous studies have identified multiple risk factors of eating disorders that are related to emotion regulation and coping strategies in the Western context. However, most of these studies failed to describe any kind of hierarchy or interaction between risk factors that co-occur. To address this knowledge gap, the present study investigated a broad range of risk factors from the perspective of emotion regulation and then used a decision tree classification method to screen for EDs among young women in China. Results showed that body image inflexibility, psychological distress, and body dissatisfaction were the primary classifiers for Chinese women at high risk of EDs.

**Supplementary Information:**

The online version contains supplementary material available at 10.1186/s40337-022-00545-6.

## Background

Eating disorders (EDs), mental illnesses that involve disturbances in body image and eating behaviors, are prevalent globally and represent a threat to public health [1, 2]. Disordered eating behaviors, ranging from severe food restriction (as evidenced in anorexia nervosa) to binge eating (as evidenced in bulimia nervosa and binge eating disorders), can result in serious health consequences, disability, and even death [3]. The prevalence of EDs more than doubled between 2005 and 2018, from 3.5 to 7.8% [4, 5]. Though research on EDs in Asian countries did not begin until the 1990s, it has since become a recognized form of psychopathology among young Asian females [6] with prevalence rising over time, especially in China [7, 8]. A recent national study showed that the prevalence of EDs is 7.04% in females aged 12–50 years in China, which is comparable to other global regions [9]. Given China’s large population base, more attention should be paid to its rate of EDs, especially in those individuals who are known to be at high risk.

Adolescent girls and young women have the highest risk of developing EDs [10, 11]. For example, Yao and colleagues [9] found that those aged 21–25 had the highest prevalence of EDs in China. Consistent with this, Stice and colleagues [11] claimed that the peak age of onset of EDs in women is 16–20 years. Ample evidence has shown that females in this age group experience more vulnerabilities [12] associated with EDs, such as body-related social comparison [13], body dissatisfaction [14], emotional difficulties [15], and psychological distress [5].

A growing body of research has focused on emotion regulation (ER) theory [16–18] and the effects of maladaptive coping strategies on eating behaviors [19, 20]. For example, loss of control over eating, a core symptom of bulimia nervosa and binge-eating disorder, is considered a strategy for coping with negative emotions [21]. More specifically, people who lose control over eating tend to think that eating can distract them from or comfort them in response to negative emotions [22]. Emotional overeating, defined as overeating in response to negative emotions, has been found to be a strong predictor of overeating-related symptoms (e.g., binge eating) in EDs [23]. Taken together, this evidence suggests that individuals who lack ER skills may develop maladaptive eating behaviors in response to negative emotions, thus contributing to the development and maintenance of EDs [24]. From this perspective, well-recognized risk factors for EDs such as body dissatisfaction and psychological distress [25, 26] may further contribute to negative emotions and trigger disordered eating behaviors through maladaptive strategies such as emotional overeating and loss of control over eating [27].

Symptoms of anorexia nervosa involve difficulty in dealing with emotional responses (e.g., fear) to weight gain, and preoccupation with food restriction and weight control [28]. These symptoms are closely related to the concept of psychological inflexibility (i.e., the inability to behave flexibly [29]), and some research has characterized anorexia nervosa as a disorder of psychological inflexibility [30]. Two specific types of psychological inflexibility have been proposed: body image inflexibility [31] and eating inflexibility [32]. Body image inflexibility refers to the unwillingness to experience negative thoughts and emotions about one’s appearance [33]. In a recent longitudinal study, body image flexibility emerged as the strongest predictor of ED psychopathology [34]. Other research has found that the link between disordered eating cognitions (e.g., fear of weight gain) and ED symptoms is established, in part, through body image inflexibility [35]. Eating inflexibility refers to an eating pattern that rigidly adheres to eating-related rules without respecting internal needs [36]. It has been shown to be significantly and positively associated with EDs, dietary restraint, picky eating, body image inflexibility, and overall psychological distress [32, 37].

Given the cross-cultural vulnerability of young females to develop EDs, research exploring new methodologies is needed to explain and stratify the various levels of risk among this group. The existing literature mainly uses regression-based methodologies, which involve rigid assumptions (e.g., linearity, homoscedasticity, independence, and normality) [38, 39] that are usually not met in studies of eating psychopathology (e.g., normality [40–42]). In other words, regression-based methodologies may be inappropriate when there are non-linear relationships or a large number of potential interactions among risk factors, making the predictive power of each factor on ED risk unclear. It is therefore recommended that researchers pay attention to the concurrency and interactions among risk factors [43] as a way to try to understand psychopathology as an effect of all factors working together [44].

To address the concurrency and interactions among variables, machine learning has been used to explore the risk factors for several mental disorders [45–47]. In a 2-year longitudinal study, Haynos and colleagues [47] demonstrated the usefulness of machine learning in predicting ED symptoms. Specifically, decision tree-based models can provide a sequential and hierarchical combination of variables with which to optimize a screen, thereby avoiding the introduction of researcher bias [47, 48].

The existing literature contains few studies of disordered eating that use machine learning methods, and those that do exist were conducted in Western societies. For example, Linardon and colleagues [48] explored the roles of three different eating patterns (e.g., rigid, flexible, and intuitive) in predicting recurrent binge eating and found intuitive eating to be the most important factor. Another study used a decision tree method to predict the onset of EDs and found that BMI, overeating, and body dissatisfaction were the three strongest predictors of EDs [43]. However, to the best of our knowledge there has been no machine learning-based research that included eating or body image inflexibility as independent variables or considered the relationship between ER and maladaptive eating behaviors. Furthermore, there is no research to date using machine learning to explore ED risk factors among Chinese women. Considering that culture is significant to the cause and expression of EDs [49], more research should be conducted in China. Thus, by using a machine learning approach, the present study aimed to fill this gap in the literature by including a range of well-established ED risk factors from the ER perspective to identify young women in China who might be at high risk of EDs.

## Methods

### Participants and procedure

The data used in the current study were from a project approved by the Institutional Review Board of the Chinese University of Hong Kong, Shenzhen, involving participants recruited from Hunan Agriculture University. The survey was conducted using paper and pencil. Psychology teachers at the university introduced the study to their 1412 first- and second-year undergraduate students during class time and invited them to participate. During data collection, all participants provided informed consent and were reminded of survey independence and authenticity. In addition, two attention check questions were used to ensure the quality of the responses. After removing those who did not provide informed consent or failed the two attention check questions, a total of 1,065 undergraduate students were eligible. Of these, our study focused on the women (*n* = 830, 77.9%), the ages of whom ranged from 18–23 years (mean = 18.91, *SD* = 0.95).

### Measures

#### Independent variables

The present study aimed to include a range of ER-related risk factors to identify young women in China at high risk of EDs.

##### Body Mass Index (BMI)

BMI was derived from self-reported height and weight and ranged from 15.22 to 31.22 kg/m^2^ with a mean of 20.16 kg/m^2^ (*SD* = 2.39).

##### Psychological distress

The Chinese version of the 6-item Kessler Scale (K6) was used to assess psychological distress. The scale consists of 6 items on a 5-point Likert scale from 0 (all of the time) to 4 (none of the time) in regards to psychological distress [50]. Sample items include *“During the past 4 weeks, how much of the time did you feel hopeless?”* The Cronbach’s α coefficient (i.e., internal consistency reliability; 0.84) and the test–retest Spearman correlation coefficient (0.79) have been shown to be acceptable in Chinese undergraduate students [51]. The Cronbach’s α of the K6 in the current study was 0.90.

##### Eating inflexibility

To assess eating inflexibility, the Chinese version of the Inflexible Eating Questionnaire (C-IEQ) was used. The IEQ consists of 11 items on a 5-point Likert scale from 1 (fully disagree) to 5 (fully agree). Sample items include *“When I cannot follow my eating plan, I feel very anxious (or nervous).”* [32]. The Chinese version of the IEQ [52] demonstrated acceptable internal consistency reliability with a Cronbach’s α of 0.87 in undergraduate students [53]. Furthermore, significant correlations between the C-IEQ and other related constructs (e.g., ED symptomatology, body image inflexibility) indicated convergent validity of this measure [52]. The Cronbach’s α of the C-IEQ in the current study was 0.90.

##### Body image inflexibility

Body image inflexibility was assessed by the Chinese version of the Body Image Acceptance and Action Questionnaire short form (C-BI-AAQ-5). The BI-AAQ-5 is a 5-item abbreviated scale of the 12-item BI-AAQ [54]. Statements such as *“Worrying about my weight makes it difficult for me to live a life that I value”* were evaluated with a 7-point Likert scale from 1 (never true) to 7 (always true) [54]. Similar to previous studies of body image inflexibility [55, 56], to calculate the total score we averaged the item responses rather than reverse scoring all items and then averaging the responses. In previous research, internal consistency reliability was acceptable (Cronbach’s α = 0.89), and strong correlations with related constructs (e.g., psychological inflexibility and body dissatisfaction) showed convergent validity in undergraduate students [57]. The Cronbach’s α of the C-BI-AAQ-5 in the current study was 0.92.

##### Body dissatisfaction

The body dissatisfaction subscale of the Eating Disorder Inventory (EDI-BD) was used to assess body dissatisfaction. The EDI-BD contains 9 items such as *“I think that my thighs are too large.”* Items are rated on a 6-point scale from 1 (never) to 6 (always) [58]. The Chinese Version of EDI demonstrated acceptable reliability and validity in undergraduate students [59]. In the current study, Cronbach’s α for the EDI-BD subscale was 0.89.

##### Emotional overeating

The Emotional Overeating subscale of Chinese Adult Eating Behavior Questionnaire (C-AEBQ) was used to measure emotional overeating. It contains 5 items such as “*I eat more when I am upset*” which were evaluated on a 5-point Likert scale from 1 (fully disagree) to 5 (agree). Previous studies showed the C-AEBQ had acceptable internal consistency reliability and convergent and divergent validity in Chinese undergraduate students [60]. Cronbach’s α of the Emotional Overeating subscale in the current study was 0.94.

##### Loss of control over eating

The Chinese version of Loss of Control Over Eating Scale-Brief (C-LOCES-B) was used to assess loss of control over eating. The LOCES-B contains 7 items from the 24-item LOCES, both of which have been validated with Cronbach’s α of 0.96 and 0.93, respectively [21]. Statements such as *“I felt helpless about controlling my eating”* were scored on a 5-point Likert scale ranging from 1 (never) to 5 (always). The C-LOCES-B showed acceptable internal consistency reliability with a Cronbach’s α of 0.92 in Chinese undergraduate students [61]. Cronbach’s α of the C-LOCES-B in the current study was 0.92.

#### Dependent variable

##### Risk of eating disorders

The Chinese version of Short Form of the Eating Disorder Examination Questionnaire (EDE-QS) [62] was used to measure the risk of EDs. The EDE-QS consists of 12 items with response options ranging from 0 to 3 days within the past 7 days [63]. The Chinese version of EDE-QS showed good psychometric properties in Chinese university students [62]. In the current study, Cronbach’s α of the EDE-QS was 0.90. A total score of 15 or above on the EDE-QS showed good sensitivity and specificity to screen individuals with EDs [64]. In the current sample, 133 females (16%) had EDE-QS scores of 15 or above, and thus were considered at high risk of developing EDs.

### Data analytic strategy

#### Dealing with missing values

In these data, the percentage of missing values across study variables ranged from 0.12% (*loss of control over eating*) to 2.29% (*eating inflexibility*). To make full use of the dataset, these missing values were handled by imputation rather than abandonment. Imputation of missing values was performed by *IterativeImputer* [65] with the *fancyimpute* package [48] of Python. The missing values were replaced with predictions from regression, and the imputation process was performed for each feature in an iterative fashion for a given number of rounds. The results of the final imputation round are returned.

#### Balancing data

Our data were imbalanced with a smaller proportion of positive cases (16.02%, 133 out of 830 samples) than negative ones (83.98%, 697 out of 830 samples). Directly training a model with imbalanced data can result in good performance for the majority class but poor performance for the minority class. For the present dataset, it would result in a model that underperforms when applied to groups at high risk of EDs [66, 67]. To handle imbalanced data in our classifications, we adopted data-level approaches, such as over- and under-sampling methods, which preprocess the imbalanced data before model training begins. The synthetic minority oversampling technique (SMOTE) is one such approach that has been widely used to enhance model performance in classification tasks with imbalanced data [68, 69]. SMOTE, as proposed by Chawla and colleagues [70], creates synthetic data for the minority class. It works by finding k-nearest neighbors from random data in the minority class and then synthesizes new data between the randomly picked data and its k-nearest neighbors. It has also proven efficient at improving model performance in decision tree models [71, 72]. Therefore, after splitting the training and testing data, the training set was balanced with SMOTE prior to model training.

In our task, 70% of the data (*n* = 581 of 830) was used for training and 30% (*n* = 249 of 830) was used for testing. The original training dataset had 93 (16.01%) positive samples and 488 (83.99%) negative samples. After SMOTE, 395 synthetic positive samples were added to the training set to yield a final *n* equal to that of the negative samples.

#### Decision tree construction

We performed decision tree classification using the *sklearn* package of Python [73]. The decision tree algorithm operates as follows. First, all samples are contained in a root node, which is then partitioned according to the best attribute (selected using Gini or Entropy). After selecting the best attribute, the algorithm then selects a split point that will maximize the purity of the resulting groups, and the dataset is partitioned into two new nodes. For each new node, the procedure is repeated recursively until no further splitting can improve the model’s predictive accuracy [74]. Some branches in the tree may even be pruned (removed) to improve predictive accuracy.

The development of a decision tree model with preprocessed data can be divided into two parts: model training and model testing. Before training, all the samples are first randomly split into two subsets, training and testing, with a defined size ratio. In the present study the size ratio was set at 7:3 (training to testing). The decision tree classification algorithm was applied to the training set to build the model. The tree was then pruned to avoid overfitting these data, and the maximum number of leaf nodes allowed in the tree was restricted. By limiting the number of leaf nodes, the number of attributes selected in the tree and the depth of the tree can be well controlled.

Model testing is the evaluation of the model’s performance when applied to the testing dataset. Each sample in the testing set was tested against the decision tree, tracing a path from the root to a specific leaf node which returned the predicted class for the sample [75]. If the predicted class for a sample was the same as the actual class, then the prediction was scored as correct for that sample. Sensitivity of the model was determined as the proportion of positive tuples (samples classified at high risk of EDs) that were correctly identified, and specificity was the proportion of negative tuples (samples classified at low risk of EDs) that were correctly identified.

## Results

### Preliminary analyses

Descriptive statistics for the entire dataset and ED risk classifications are presented in Table 1. Pearson correlations among the independent variables are presented in Additional file 1: Table S1. The following features were found at higher rates among samples classified as high-risk than in those classified as low-risk: higher BMI, eating inflexibility, psychological distress, loss of control over eating, body image inflexibility, body dissatisfaction, and emotional overeating.

**Table 1:** Comparison of groups on study variables

	Eating disorder risk classification						
	Total sample (*n* = 830)		No (*n* = 697)		Yes (*n* = 133)		
Variable	*M*	*SD*	*M*	*SD*	*M*	*SD*	*d* [95% CI]
BMI	20.16	2.40	20.04	2.41	20.82	2.20	0.33 [0.14, 0.51]
Eating inflexibility	30.70	7.11	29.91	6.87	34.88	6.91	0.72 [0.53, 0.91]
Psychological distress	11.71	4.32	11.00	3.87	15.45	4.64	1.11 [0.92, 1.31]
Loss of control over eating	13.34	5.51	12.34	4.88	18.59	5.68	1.25 [1.05, 1.45]
Body image inflexibility	11.59	6.30	10.11	5.10	19.35	6.29	1.74 [1.53, 1.95]
Body dissatisfaction	34.60	9.13	33.61	8.79	39.79	9.16	0.70 [0.51, 0.89]
Emotional over eating	2.18	0.92	2.06	0.88	2.83	0.87	0.88 [0.69, 1.07]

*M* = mean, *SD* = standard deviation, *d* = Cohen’s d, CI = confidence interval. The classification of “Yes” stands for groups classified with high risk of EDs, and “No” stands for groups classified with low risk of EDs

### Decision tree classification model construction

Body image inflexibility, psychological distress, and body dissatisfaction were identified as important classifiers for identifying individuals at high risk of EDs (i.e., having EDE-QS scores equal to or greater than 15). As an indicator of relative importance between classifiers, feature importance was then calculated for each of the three major classifiers. Feature importance scores were 0.81, 0.15, and 0.04, for body image inflexibility, psychological distress, and body dissatisfaction, respectively.

**Fig. 1:**
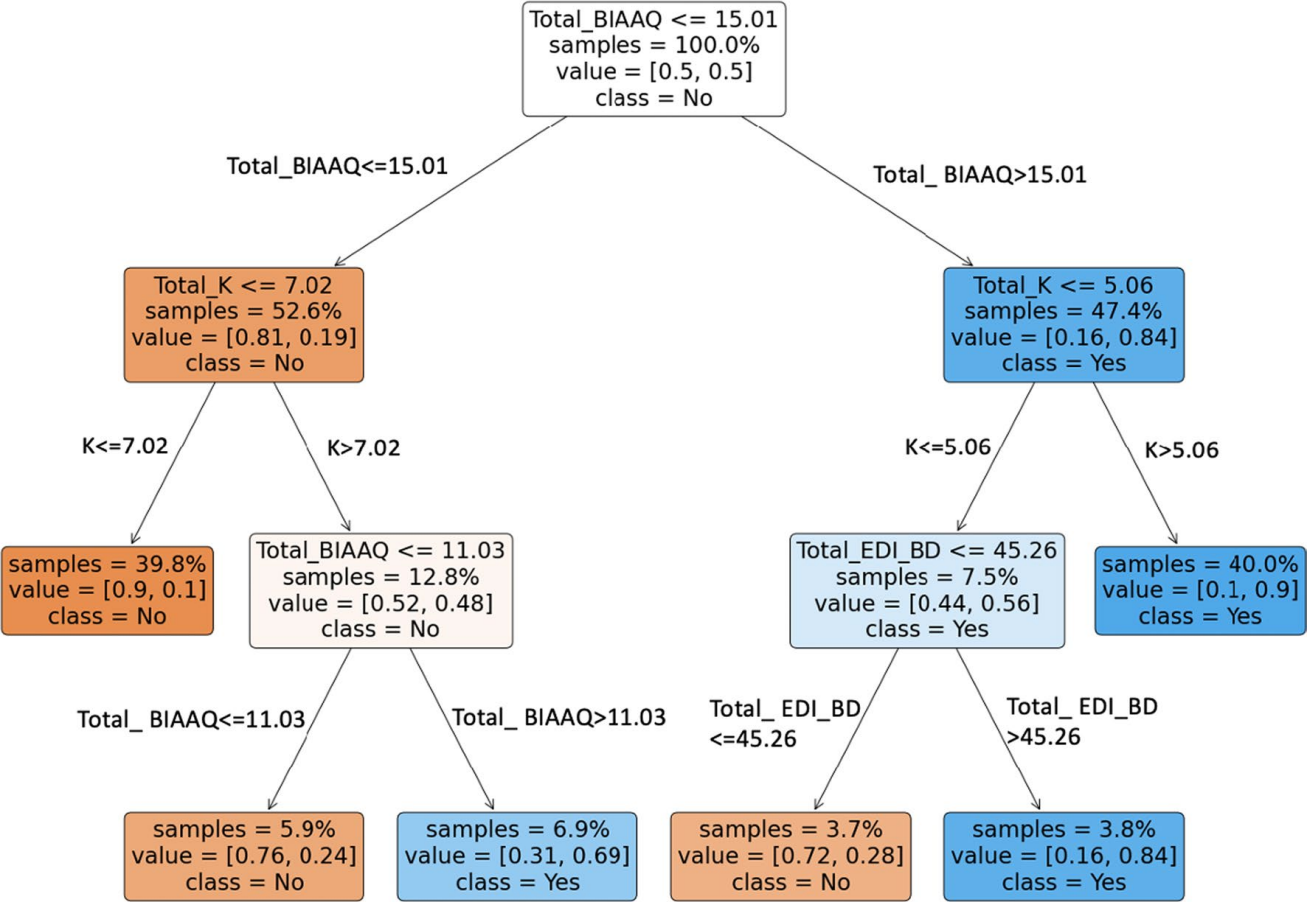
Decision tree for classifying at-risk of EDs. *Note*: Figure shows the classification tree for at-risk of EDs based on the training subsample of the overall dataset (*n* = 581 of 830). Total_BIAAQ = total score of Body Image Acceptance and Action Questionnaire. Total_K = total score of Kessler Scale to assess psychological distress. Total_EDI_BD = Body Dissatisfaction subscale of the Eating Disorder Inventory. For each internal node, the first line refers to a decision rule with a selected attribute. For example, the root node indicates a decision rule that the attribute *body image inflexibility* is smaller than or equal to 15.01. For a node with branches, its left child node follows the decision rule in the parent node, whereas its right child follows the complement of the decision rule. The second line of each internal node indicates the percentage of samples involved in this node. The third line refers to the percentages of positive samples (i.e., samples at-risk of EDs) and that of negative samples (i.e., samples without at-risk of EDs) within each node. The shade of color refers to the purity of each node, implying the extent of a mixture of groups for a subset of samples. The dark color means most samples belong to one group. Lastly, *class* in each box indicates whether high risk of EDs is more prevalent in a node. Blue boxes with *class* = *yes* indicate at-risk of EDs is more prevalent, whereas orange boxes with *class* = *no* indicate the subgroups contain more people with low risk of EDs, based on EDE-QS scores

As shown in Fig. 1, participants were first partitioned by body image inflexibility scores as measured by the BI-AAQ-5. Participants with higher body image inflexibility scores ($>$ 15.01) constituted 47.4% of the overall training samples, and 84% of these participants were classified at high risk of EDs. These participants were further partitioned by psychological distress scores as measured by the K6. Those with higher psychological distress scores (> 5.06) were further classified at high risk of EDs. Those with lower psychological distress scores ($\le$ 5.06) were further partitioned by body dissatisfaction scores as measured by the EDI-BD. Among these participants, those with higher body dissatisfaction scores ($>$ 45.26) tended to be at high risk of EDs, while the others were not.

Participants with lower body image inflexibility scores ($\le$ 15.01) were partitioned and classified in a different way. Those with lower psychological distress scores ($\le$ 7.02) were at low risk of EDs. Individuals with higher scores (> 7.02) were further partitioned by body image inflexibility. Those with lower body image inflexibility scores ($<$ 11.03) were at low risk of EDs, while those with relatively higher body image inflexibility scores ($>$ 11.03 and $\le$ 15.01) were at high risk.

Overall, participants with higher body image inflexibility scores, higher psychological distress scores, and/or higher body dissatisfaction scores were more likely to be categorized at high risk of EDs.

### Decision tree classification model evaluation

Similar to a previous study [48], the present model was evaluated according to its sensitivity and specificity. Sensitivity reflects the probability that the model will correctly include individuals at high risk of EDs, and specificity reflects the probability that it will correctly exclude those that are not at high risk of EDs. When classifying individuals from the test subset of data (*n* = 249 of 830) the model had a sensitivity of 0.88 and a specificity of 0.85. That is, the model performs slightly better in correctly identifying people at high risk of EDs (88%, *n* = 40) than for classifying those not at high risk (85%, *n* = 209). To further validate the evaluation results across the entire dataset, a random sampling method was adopted. By randomly selecting 30% of the overall dataset, five different times, the average sensitivity was 0.85 and the average specificity was 0.86, which aligns with the evaluation of the test subsample.

**Fig. 2:**
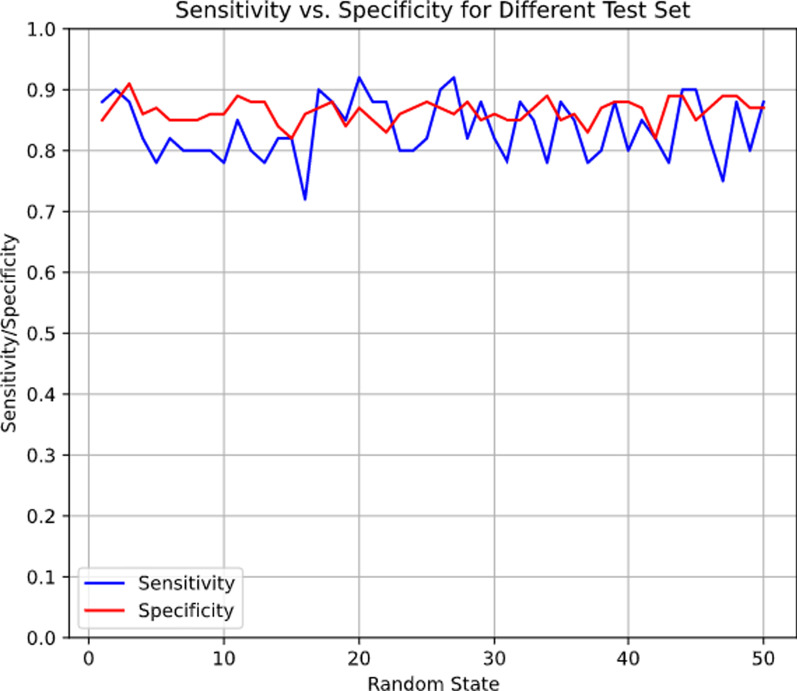
Sensitivity and specificity for different test set. *Note*: The random state determines the randomness in the split of training set and test set. Therefore, different random states correspond to different test sets. Our proposed model was evaluated by each test set and the test sensitivity and specificity were recorded for each test set. In the figure above, the blue curve represents the change of test sensitivity with different test sets and the red curve represents the change of test specificity with different test sets

Regarding model complexity, seven attributes were initially taken into consideration and the model ultimately selected three of them. The minimum number of samples contained in a single node was 36 (3.7% of 976 training samples, including 395 synthetic training samples and 581 original training samples). Moreover, we generated 50 different test sets by changing the random seed used to split the training and test sets and used these to further evaluate the proposed model. The mean sensitivity across the 50 test sets was 0.84 (*SD* = 0.05) and mean specificity was 0.86 (*SD* = 0.02). As shown in Fig. 2, these results demonstrate the sufficiency of our samples for yielding a stable model.

## Discussion

With machine learning, the current study used a decision tree to identify young women in China who may be at high risk of developing an ED. The strengths of this study include its ER framework to examine several common risk factors for EDs in a non-clinical sample of young women in China. Furthermore, the decision tree method allows for deeper exploration of these variables concurrently and how they may interact in relation to ED risk among young women in China. The findings add to our understanding of the role of body image inflexibility in detecting EDs in the Chinese context, which is critical because most existing research has been conducted in Western cultures. Furthermore, distinctive interactions between the major classifiers show that in young women with a high level of body image inflexibility, elevated psychological distress could amplify the risk of EDs. These findings further suggest that increasing body image flexibility may improve tolerance to psychological distress and thus lower the risk of EDs among young Chinese women.

The present findings have several implications for the intervention and prevention of EDs in non-clinical young women in China. First, body image inflexibility was the most significant factor for detecting EDs in the current sample. This is consistent with prior studies in the United States that demonstrated a key role of body image flexibility and body image inflexibility in the development of EDs [35, 76]. For example, intervention studies indicated that body image flexibility was the strongest predictor of ED symptomatology [34, 76]. In addition, as body image flexibility and body image inflexibility have been proposed as variables linking negative emotion [77], negative body image [78], and negative cognitions [35] with EDs, it seems necessary to incorporate body image inflexibility with theoretical models under emotion and cognition frameworks to better understand the psychopathology of EDs. Body dissatisfaction was rated less important than body image inflexibility or psychological distress by the present model; thus, future research into ED prevention and intervention should prioritize body image flexibility/inflexibility and psychological distress. This is in line with the common practice of treating EDs with Acceptance and Commitment Therapy, in which body image flexibility is promoted as a major adaptive emotional mechanism to reduce ED psychopathology [79].

When introducing psychological distress into the decision tree model, the effects and interactions between psychological distress and body image inflexibility were found to be distinctive. Of the group with high body image inflexibility and high psychological distress, 90% were found to be at high risk of EDs, indicating that elevated psychological distress may amplify the risk of EDs in individuals who have a high level of body image inflexibility. On the other hand, participants with low body image inflexibility and psychological distress were classified as having low risk of EDs. Comparing the two thresholds of psychological distress under different levels of body image inflexibility, the present findings suggest that a lower level of body image inflexibility may reduce the impact of psychological distress on ED risk: those with low body image inflexibility were generally at lower risk of EDs regardless of the level of psychological distress. This makes sense given that greater body image flexibility can help people accept negative emotions and thoughts about their bodies, which may in turn promote more adaptive behaviors when dealing with such emotions [80]. Previous research has shown a negative correlation between psychological flexibility and disordered eating [81, 82], and a protective effect of psychological flexibility in relation to stress and mental health [83]. Still, more research is needed to probe the interaction between these factors and their role in EDs.

It is also worth noting that the group with high levels of psychological distress was further partitioned by body image inflexibility into two groups (with either high or low risk of EDs), indicating that mid-level body image inflexibility concurrent with high-level psychological distress may also be associated with high risk of EDs. Nuanced interactions such as this could provide a framework for clinical practice involving a variety of mental health conditions. For example, practitioners may focus on reducing both body image inflexibility and psychological distress for individuals with high body image inflexibility, whereas they may focus more on targeting psychological distress for individuals with low body image inflexibility. More research is needed to investigate the interactions and thresholds of these two factors in other datasets and using different intervention outcomes.

As a third-level classifier, body dissatisfaction separates those with high body image inflexibility and low psychological distress into two further nodes. In the high node (body dissatisfaction > 45.26, as compared to the sample mean of 34.60), 84% of the group were classified as high-risk. This is consistent with numerous studies showing positive correlations between body dissatisfaction and ED psychopathology [14, 84], although the sample size identified by this node was relatively small (only 3.8% of the total sample). Even though it is a well-acknowledged risk factor for EDs, body dissatisfaction contributed less to the current decision tree model than expected; its feature importance was only 4%. This differed from the results of another machine learning-based study showing that body dissatisfaction was the most important classifier of EDs among variables including, but not limited to, depressive symptoms, negative affectivity, and dietary restraint [11]. A reason why body dissatisfaction was less important in the current model could be explained by interaction effects between body dissatisfaction and body image inflexibility. Body image inflexibility has been identified as a mediator between body dissatisfaction and eating disorder symptoms in several studies [78, 85]. In mediation analysis, the direct effects of body dissatisfaction on disordered eating dropped significantly after adding body image inflexibility as a mediator [85]. Thus, analogous to the effects in the mediation model, it could be that the inclusion of body image inflexibility weakens the predictive power of body dissatisfaction in the current decision tree model. However, this hypothesis should be tested in samples from other cultures or populations.

The present findings may be beneficial for practitioners to understand and intervene in ED symptomatology in non-clinical young women in China. For instance, when designing large-scale and cost-effective prevention/intervention programs to reduce ED symptomatology among young women in China, targeting factors may be prioritized to include the three risk factors highlighted in the current study. However, caution should be taken when interpreting the present findings. First, to reduce model complexity, some of our initial variables were not used in the final decision tree (BMI, emotional overeating, loss of control over eating, and eating inflexibility). This does not necessarily mean that these variables are not related to ED risk, but rather that they convey less explanatory and interactive power than others in this particular set of variables and data. Moreover, the results, especially the thresholds of the classifiers, should be considered provisional until validated by future studies. Finally, based on the sensitivity analysis (see Additional file 1) with another sample of non-clinical young women in which SCOFF [86] was used as the screening tool, the results consistently showed that body image inflexibility was the most important risk factor, supporting the robustness of the current findings on body image inflexibility.

The current study is not free from limitations. First, the sample only consisted of young women from a university population. Further exploration of larger and more diverse samples is needed to be able to generalize to different populations (e.g., adolescent girls and adult women). Second, the study’s cross-sectional design limits our ability to draw conclusions about causal relationships between study variables. Well-designed prospective research is needed to confirm these exploratory findings. Thirdly, the EDE-QS can only screen participants at risk of EDs in general; in other words, it cannot distinguish specific EDs such as anorexia nervosa, bulimia nervosa, or binge-eating disorder. Thus, the current results cannot be generalized to patients with a specific ED diagnosis, and we expect studies involving patients with EDs (e.g., [87]) to further explore the risk factors of specific types of EDs in the Chinese context.

## Conclusions

This is the first study to use a decision tree classification analysis in the Chinese context to explore interactions between ER-related factors and ED risk. Several conclusions were drawn from a decision tree built upon data with three major classifiers: body image inflexibility, psychological distress, and body dissatisfaction. Among them, body image inflexibility was most predictive of high ED risk, which is consistent with a number of other studies. Furthermore, novel interactions between body image inflexibility and psychological distress were found in relation to ED risk. These findings could be further extended to identify potentially high-risk groups in order to guide ED prevention measures among young Chinese women. Future studies are necessary to test the interactive hypotheses and improve prediction accuracy for the assessment, diagnosis, and intervention of EDs in the Chinese context.

## Supplementary Information

**Additional file 1.** Pearson correlation analysis and sensitivity analysis.

## Data Availability

The datasets used and/or analyzed during the current study are available from the corresponding author on reasonable request.

## Abbreviations

EDs: Eating disorders
ER: Emotion regulation
C-AEBQ: Chinese translation of the Adult Eating Behavior Questionnaire
IEQ: Inflexible Eating Questionnaire
EDI: Eating Disorder Inventory
BI-AAQ: Body Image Acceptance and Action Questionnaire
LOCES-B: Loss of Control Over Eating Scale-Brief
