# Supplementary material for: Using machine learning to explore core risk factors associated with the risk of eating disorders among non-clinical young women in China: A decision-tree classification analysis
Source: J Eat Disord. 2022 Feb 10;10:19. doi: 10.1186/s40337-022-00545-6 (PMC8832719; doi:10.1186/s40337-022-00545-6)
Supplement: Supplementary file 1 — Additional file 1. Pearson correlation analysis and sensitivity analysis. [file 40337_2022_545_MOESM1_ESM.docx]

**Table 1S.**

*Pearson correlation between independent variables*

| Variable | 1 | 2 | 3 | 4 | 5 | 6 | 7 |
| --- | --- | --- | --- | --- | --- | --- | --- |
| 1.BMI | - | -0.002  [-0.17, 0.17] | -0.21 * [-0.36, -0.04] | 0.08  [-0.09, 0.25] | 0.19 * [0.02, 0.35] | 0.47 *** [0.32, 0.59] | 0.05  [-0.12, 0.22] |
| 2.Eating Inflexibility | 0.003  [-0.07, 0.08] | - | 0.05  [-0.12, 0.22] | 0.02  [-0.15, 0.19] | 0.18 * [0.01, 0.34] | 0.25 ** [0.09, 0.41] | 0.13  [-0.05, 0.29] |
| 3.Psychological Distress | -0.03  [-0.1, 0.05] | 0.06  [-0.01, 0.14] | - | 0.4 *** [0.25, 0.53] | 0.31 *** [0.15, 0.46] | -0.21 * [-0.37, -0.04] | 0.39 *** [0.24, 0.53] |
| 4.Loss of Control Over Eating | 0.14 *** [0.06, 0.21] | 0.13 *** [0.05, 0.2] | 0.42 *** [0.36, 0.48] | - | 0.36 *** [0.2, 0.5] | 0.12  [-0.05, 0.28] | 0.45 *** [0.3, 0.58] |
| 5.Body Image Inflexibility | 0.28 *** [0.21, 0.35] | 0.24 *** [0.17, 0.31] | 0.34 *** [0.27, 0.41] | 0.52 *** [0.47, 0.58] | - | 0.34 *** [0.18, 0.49] | 0.30 *** [0.14, 0.45] |
| 6.Body Dissatisfaction | 0.52 *** [0.46, 0.57] | 0.07  [-0.01, 0.14] | 0.14 *** [0.07, 0.21] | 0.3 *** [0.23, 0.37] | 0.39 *** [0.32, 0.45] | - | 0.09  [-0.08, 0.26] |
| 7.Emotional Overeating | 0.10 * [0.02, 0.17] | 0.12 ** [0.04, 0.19] | 0.26 *** [0.19, 0.33] | 0.44 *** [0.38, 0.5] | 0.43 *** [0.37, 0.49] | 0.15 *** [0.08, 0.22] | - |

*Note:* The upper-right data represents the Pearson correlation estimates and confidence intervals for the sub-sample at high risk of EDs. The lower-left data represents the Pearson correlation estimates and confidence intervals for the sub-sample with low risk of EDs; ^*^ *p* < .05, ^**^ *p* < .01, ^***^ *p* < .001.

**Supplementary Sensitivity Analysis**

By using a sample of female university students extracted from a project on the eating behaviors and body image of Chinese university students (see [1]), we conducted a sensitivity analysis to examine the robustness of the findings when a different EDs screening measure was used. The female university students aged from 18 to 23 years (*Mean* = 20.08, *SD* = 0.94), and their BMI, derived from self-reported height and weight, ranged from 14.30 to 36.89 kg/m^2^ (Mean = 20.70, *SD* = 3.01).

In the sensitivity analysis, the SCOFF [2] was used to screen participants at-risk of EDs (59% were screened positive on SCOFF $\geq$ 2). In line with the current study, we used the following independent variables in machine learning, namely BMI, psychological distress as measured by the 10-item Kessler Scale [3], eating inflexibility as measured by the Inflexible Eating Questionnaire [4], body image inflexibility as measured by the Body Image Acceptance and Action Questionnaire [5], body dissatisfaction as measured by the body dissatisfaction subscale of the Eating Disorder Inventory [6], and emotional overeating as measured by the emotional overeating subscale (EOE) of the Adult Eating Behavior Questionnaire [7]. However, Loss of Control Over Eating was not available in this dataset.

With the same statistical methods described in the main manuscript, results (as Figure 1S) showed that body image inflexibility, EOE, eating inflexibility, and body dissatisfaction are important features. The feature importance was .46, .22, .19, and .14. This model in classifying those at high risk of EDs in the test subsample had a sensitivity of .51 and a specificity of .77.

**Figure 1S.**

Decision Tree for Classifying high risk of EDs

**
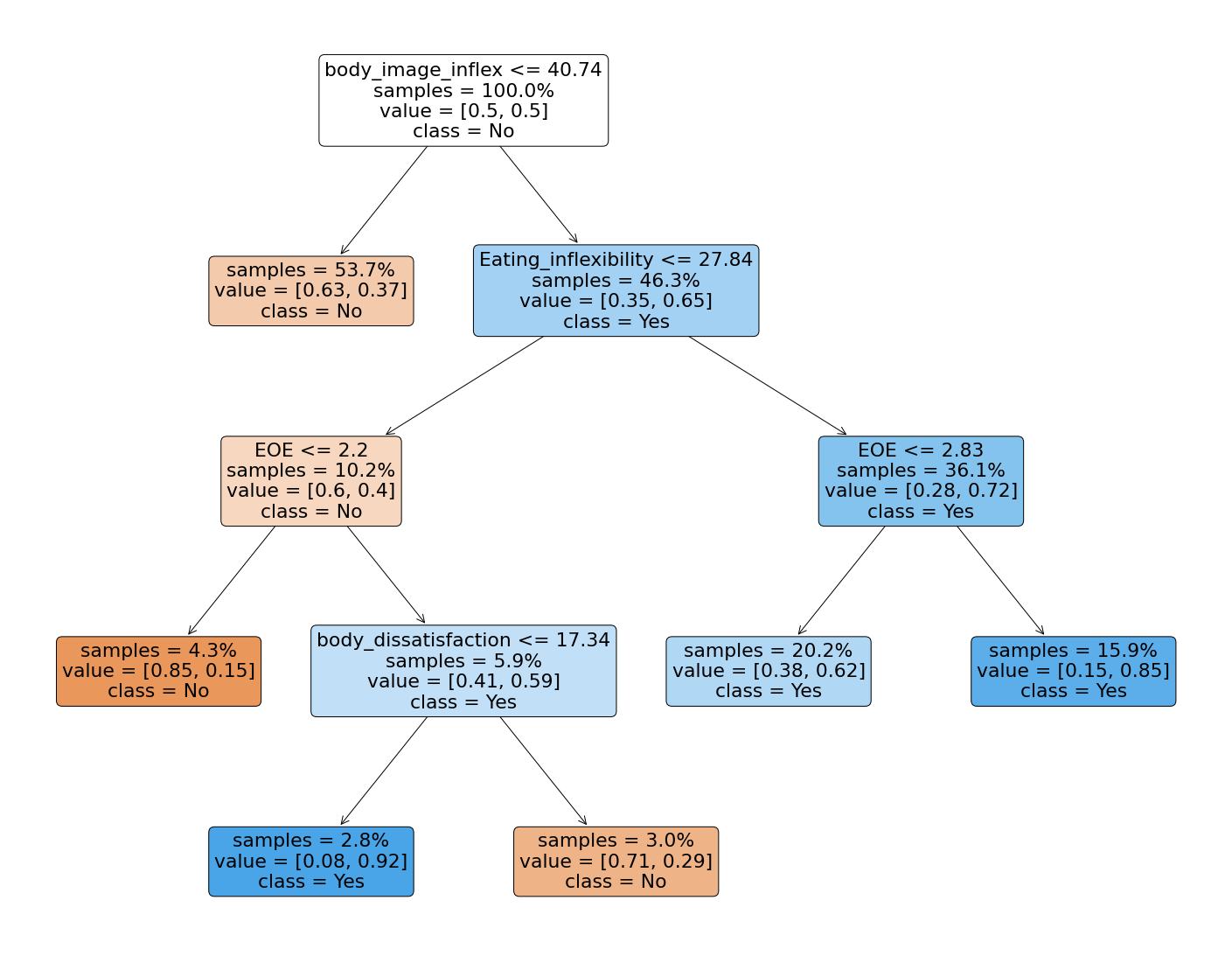
**

*Note*：Figure 1S shows the classification tree for high risk of EDs based on the training subsample of the overall dataset. For each internal node, the first line refers to a decision rule with a selected attribute. For a node with branches, its left child node follows the decision rule in the parent node, whereas its right child follows the complement of the decision rule. The second line of each internal node indicates the percentage of samples involved in this node. The third line refers to the percentages of positive (high risk of EDs) samples and that of negative (low risk of EDs) samples within each node. The shade of color refers to the purity of each node, implying the extent of a mixture of groups for a subset of samples. The dark color means more samples belong to one group. Lastly, *class* in each box indicates whether high risk of EDs is more prevalent in the node. Blue boxes with *class = yes* indicate high risk of EDs is more prevalent, whereas orange boxes with *class = no* indicate the subgroups contain more people with low risk of EDs, based on SCOFF scores.

**References**

1. He J, Sun S, Fan X: **Validation of the 12-item Short Form of the Eating Disorder Examination Questionnaire in the Chinese context: confirmatory factor analysis and Rasch analysis**. *Eat Weight Disord* 2021, **26**(1):201-209.<https://doi.org/10.1007/s40519-019-00840-3>

2. Morgan JF, Reid F, Lacey JH: **The SCOFF questionnaire: assessment of a new screening tool for eating disorders**. *Bmj* 1999, **319**(7223):1467-1468 https://doi.org/10.1136/bmj.319.7223.1467

3. Kessler R, Mroczek D: **Final versions of our non-specific psychological distress scale**. *Ann Arbor, MI: Survey Research Center of the Institute for Social Research, University of Michigan* 1994

4. Duarte C, Ferreira C, Pinto-Gouveia J, Trindade I, Martinho A: **What makes dietary restraint problematic? Development and validation of the Inflexible Eating Questionnaire**. *Appetite* 2017, **114**:146-154 https://doi.org/10.1016/j.appet.2017.03.034

5. Sandoz EK, Wilson KG, Merwin RM, Kellum KK: **Assessment of body image flexibility: the body image-acceptance and action questionnaire**. *Journal of Contextual Behavioral Science* 2013, **2**(1-2):39-48 <https://doi.org/10.1016/j.brat.2005.06.006>

6. Leung F, Wang J, Tang CW-y: **Psychometric properties and normative data of the Eating Disorder Inventory among 12 to 18 year old Chinese girls in Hong Kong**. *Journal of psychosomatic research* 2004, **57**(1):59-66 <https://doi.org/10.1002/(sici)1098-108x(199703)21:2><187::aid-eat10>3.0.co;2-#

7. He J, Sun S, Zickgraf HF, Ellis JM, Fan X: **Assessing appetitive traits among Chinese young adults using the adult eating behavior questionnaire: Factor structure, gender invariance and latent mean differences, and associations with BMI**. *Assessment* 2019:1073191119864642 https://doi.org/10.1007/s40519-019-00840-3
